# Supplementary material for: Monitoring the trends of Angiostrongylus cantonensis infection in humans and Pomacea spp. Snails in Dali, Yunnan, China, 2007–2021
Source: PLoS Negl Trop Dis. 2025 May 5;19(5):e0013065. doi: 10.1371/journal.pntd.0013065 (PMC12052120; doi:10.1371/journal.pntd.0013065)
Supplement: S1 Table — (DOCX) [file pntd.0013065.s001.docx]

Supplemental Table 1. GenBank accession numbers and corresponding references for the COI and ITS2 phylogenetic tree.

| No | Species | Name | Accession No. | Gene | Location | Host |
| --- | --- | --- | --- | --- | --- | --- |
| 1 | *Angiostrongylus cantonensis* | 2020-12-28 | PP735489 | COI | Dali, China | *Pomacea spp.* |
| 2 | *An. cantonensis* | 2020-01-02.1 | OR816070 | COI | Dali, China | *Pomacea spp.* |
| 3 | *An. cantonensis* | 2020-01-02.2 | OR816071 | COI | Dali, China | *Pomacea spp.* |
| 4 | *An. cantonensis* | 2019-12-31 | OR816072 | COI | Dali, China | *Pomacea spp.* |
| 5 | *An. cantonensis* | 2018-12-25 | OR816073 | COI | Dali, China | *Pomacea spp.* |
| 6 | *An. cantonensis* | 2018-01-29 | OR816074 | COI | Dali, China | *Pomacea spp.* |
| 7 | *An. cantonensis* | 2016-11-30 | OR816075 | COI | Dali, China | *Pomacea spp.* |
| 8 | *An. cantonensis* | 24-03-2016 | OR816076 | COI | Dali, China | *Pomacea spp.* |
| 9 | *An. cantonensis* | 2015-11-12 | OR816077 | COI | Dali, China | *Pomacea spp.* |
| 10 | *An. cantonensis* | 2015-10-21 | OR816078 | COI | Dali, China | *Pomacea spp.* |
| 11 | *An. cantonensis* | 2015-10-12 | OR816079 | COI | Dali, China | *Pomacea spp.* |
| 12 | *An. cantonensis* | 2014-04-14 | OR816080 | COI | Dali, China | *Pomacea spp.* |
| 13 | *An. cantonensis* | 2014-03-13 | OR816081 | COI | Dali, China | *Pomacea spp.* |
| 14 | *An. cantonensis* | 2014-02-12 | OR816082 | COI | Dali, China | *Pomacea spp.* |
| 15 | *An. cantonensis* | 2014-01-28 | OR816083 | COI | Dali, China | *Pomacea spp.* |
| 16 | *An. cantonensis* | 2013-05-17 | OR816084 | COI | Dali, China | *Pomacea spp.* |
| 17 | *An. cantonensis* | 2013-04-11 | OR816085 | COI | Dali, China | *Pomacea spp.* |
| 18 | *An. cantonensis* | 2013-05-10 | OR816086 | COI | Dali, China | *Pomacea spp.* |
| 19 | *An. cantonensis* | 2013-02-27 | OR816087 | COI | Dali, China | *Pomacea spp.* |
| 20 | *An. cantonensis* | AP017672 | AP017672 | COI | Japan | \| Unknown \| \| --- \| |
| 21 | *An. cantonensis* | KT947978 | KT947978 | COI | Thailand | \| Unknown \| \| --- \| |
| 22 | *An. cantonensis* | KY779736 | KY779736 | COI | Cambodia | *Pomacea spp.* |
| 23 | *An. cantonensis* | KY779737 | KY779737 | COI | Vietnam | *Pomacea spp.* |
| 24 | *An. cantonensis* | KY779738 | KY779738 | COI | Vietnam | *Pomacea spp.* |
| 25 | *An. cantonensis* | MK570629 | MK570629 | COI | Spain | *Rattus rattus* |
| 26 | *An. cantonensis* | MK570630 | MK570630 | COI | Hawaii, USA | *Rattus exulans* |
| 27 | *An. cantonensis* | MK570631 | MK570631 | COI | Australia | *Rattus rattus* |
| 28 | *An. cantonensis* | MK570632 | MK570632 | COI | French Polynesia | *Rattus exulans* |
| 29 | *An. cantonensis* | NC_013065 | NC_013065 | COI | China | \| Unknown \| \| --- \| |
| 30 | *An. cantonensis* | OR177659 | OR177659 | COI | Australia | *Saimiri boliviensis* |
| 31 | *An. cantonensis* | OR177660 | OR177660 | COI | Australia | *Rattus rattus* |
| 32 | *Angiostrongylus vasorum* | NC_018602 | NC_018602 | COI | Australia | *Canis l. familiaris* |
| 33 | *Aelurostrongylus abstrusus* | NC_019571 | NC_019571 | COI | Australia | *Felis catus* |
| 34 | *Caenorhabditis elegans* | NC_001328 | NC_001328 | COI | England | None |
|  |  |  |  |  |  |  |
| No | Species | Name | Accession No. | Gene | Location | Host |
| 1 | *Angiostrongylus cantonensis* | 2013-04-17 | OR790453 | ITS2 | Dali, China | *Pomacea spp.* |
| 2 | *An. cantonensis* | 2013-05-17 | OR790454 | ITS2 | Dali, China | *Pomacea spp.* |
| 3 | *An. cantonensis* | 2014-01-26 | OR790455 | ITS2 | Dali, China | *Pomacea spp.* |
| 4 | *An. cantonensis* | 2014-03-13 | OR790456 | ITS2 | Dali, China | *Pomacea spp.* |
| 5 | *An. cantonensis* | 2014-02-12 | OR790457 | ITS2 | Dali, China | *Pomacea spp.* |
| 6 | *An. cantonensis* | 2015-11-12 | OR790458 | ITS2 | Dali, China | *Pomacea spp.* |
| 7 | *An. cantonensis* | 2015-10-12 | OR790459 | ITS2 | Dali, China | *Pomacea spp.* |
| 8 | *An. cantonensis* | 2015-10-21 | OR790460 | ITS2 | Dali, China | *Pomacea spp.* |
| 9 | *An. cantonensis* | 2016-03-24 | OR790461 | ITS2 | Dali, China | *Pomacea spp.* |
| 10 | *An. cantonensis* | 2016-01-28 | OR790462 | ITS2 | Dali, China | *Pomacea spp.* |
| 11 | *An. cantonensis* | 2018-12-25 | OR790463 | ITS2 | Dali, China | *Pomacea spp.* |
| 12 | *An. cantonensis* | 2018-01-29 | OR790464 | ITS2 | Dali, China | *Pomacea spp.* |
| 13 | *An. cantonensis* | 2019-12-31 | OR790465 | ITS2 | Dali, China | *Pomacea spp.* |
| 14 | *An. cantonensis* | 2020-01-02 | OR790466 | ITS2 | Dali, China | *Pomacea spp.* |
| 15 | *An. cantonensis* | 2020-01-09 | OR790467 | ITS2 | Dali, China | *Pomacea spp.* |
| 16 | *An. cantonensis* | 2020-12-28 | OR790468 | ITS2 | Dali, China | *Pomacea spp.* |
| 17 | *An. cantonensis* | 2021-04-01 | OR790469 | ITS2 | Dali, China | *Pomacea spp.* |
| 18 | *An. cantonensis* | AB700682 | AB700682 | ITS2 | Japan | *Rattus rattus* |
| 19 | *An. cantonensis* | AB700698 | AB700698 | ITS2 | China | *Rattus norvegicus* |
| 20 | *An. cantonensis* | AB700700 | AB700700 | ITS2 | Thailand | *Rattus norvegicus* |
| 21 | *An. cantonensis* | EU636007 | EU636007 | ITS2 | Philippines | Unknown |
| 22 | *An. cantonensis* | GQ181112 | GQ181112 | ITS2 | Spain | *Rattus rattus* |
| 23 | *An. cantonensis* | HQ540551 | HQ540551 | ITS2 | China | Unknown |
| 24 | *An. cantonensis* | JQ806757 | JQ806757 | ITS2 | Japan | Unknown |
| 25 | *An. cantonensis* | JQ806759 | JQ806759 | ITS2 | China | Unknown |
| 26 | *An. cantonensis* | KU528689 | KU528689 | ITS2 | Hawaii, USA | Unknown |
| 27 | *An. cantonensis* | OM022096 | OM022096 | ITS2 | China | *Pomacea spp.* |
| 28 | *An. cantonensis* | PP444659 | PP444659 | ITS2 | Brazil | *Lissachatina fulica* |
| 29 | *An. cantonensis* | PP796387 | PP796387 | ITS2 | China | Unknown |
| 30 | *An. cantonensis* | PQ213488 | PQ213488 | ITS2 | China | Unknown |
| 31 | *Angiostrongylus vasorum* | GU045370 | GU045370 | ITS2 | Denmark | *Vulpes vulpes* |
| 32 | *Aelurostrongylus abstrusus* | KX518353 | KX518353 | ITS2 | Germany | *Felis silvestris* |
| 33 | *Caenorhabditis elegans* | DQ232193 | DQ232193 | ITS2 | England | None |

Note: The sequences generated in this study are highlighted in red.
