# Supplementary material for: Monitoring the trends of Angiostrongylus cantonensis infection in humans and Pomacea spp. Snails in Dali, Yunnan, China, 2007–2021
Source: PLoS Negl Trop Dis. 2025 May 5;19(5):e0013065. doi: 10.1371/journal.pntd.0013065 (PMC12052120; doi:10.1371/journal.pntd.0013065)
Supplement: Attachment — Submitted filename: YHG_Response to Reviewers.docx [file pntd.0013065.s002.docx]

Thank you for reviewing our manuscript. Please see our replies below in red.

-

Dear Dr. Guo,

Thank you for submitting your manuscript to PLOS Neglected Tropical Diseases. After careful consideration, we feel that it has merit but does not fully meet PLOS Neglected Tropical Diseases's publication criteria as it currently stands. Therefore, we invite you to submit a revised version of the manuscript that addresses the points raised during the review process.

Please submit your revised manuscript within 60 days Jan 23 2025 11:59PM. If you will need more time than this to complete your revisions, please reply to this message or contact the journal office at plosntds@plos.org. When you're ready to submit your revision, log on to https://www.editorialmanager.com/pntd/ and select the 'Submissions Needing Revision' folder to locate your manuscript file.

Please include the following items when submitting your revised manuscript:

* A rebuttal letter that responds to each point raised by the editor and reviewer(s). You should upload this letter as a separate file labeled 'Response to Reviewers'. This file does not need to include responses to any formatting updates and technical items listed in the 'Journal Requirements' section below.

* A marked-up copy of your manuscript that highlights changes made to the original version. You should upload this as a separate file labeled 'Revised Manuscript with Track Changes'.

* An unmarked version of your revised paper without tracked changes. You should upload this as a separate file labeled 'Manuscript'.

If you would like to make changes to your financial disclosure, competing interests statement, or data availability statement, please make these updates within the submission form at the time of resubmission. Guidelines for resubmitting your figure files are available below the reviewer comments at the end of this letter.

We look forward to receiving your revised manuscript.

Kind regards,

Siddhartha Mahanty, M.B.B.S., M.P.H

Academic Editor

PLOS Neglected Tropical Diseases

Jong-Yil Chai

Section Editor

PLOS Neglected Tropical Diseases

Shaden Kamhawi
co-Editor-in-Chief
PLOS Neglected Tropical Diseases
orcid.org/0000-0003-4304-636XX

Paul Brindley
co-Editor-in-Chief
PLOS Neglected Tropical Diseases
orcid.org/0000-0003-1765-0002

Additional Editor Comments :

All the reviewers acknowledged the strengths of your study, particularly the large number of cases, comprehensive clinical and laboratory data and the linkage of human infections with prevalence of A. cantonensis in Pomacea spp. However, some issues with serological and molecular methodology and epidemiological design have been identified by the reviewers that should be addressed for the manuscript to be considered for publication. Please address all the reviewers' comments, paying particular attention the issues raised by reviewers 1 and 3.

-----

Reviewers' Comments:

Reviewer's Responses to Questions

Key Review Criteria Required for Acceptance?

As you describe the new analyses required for acceptance, please consider the following:

Methods:

Reviewer #1:

-Are the objectives of the study clearly articulated with a clear testable hypothesis stated? Yes
-Is the study design appropriate to address the stated objectives? Yes
-Is the population clearly described and appropriate for the hypothesis being tested? Yes
-Is the sample size sufficient to ensure adequate power to address the hypothesis being tested? Yes
-Were correct statistical analysis used to support conclusions? No
-Are there concerns about ethical or regulatory requirements being met? No

Far more detail of the ELISA employed is required. What antigen was used? - Was this an IgG or IgM ELISA? - Was it a commercial kit or in-house developed assay? - What controls were employed? - What OD cut-off was used? - Is there a reference to any validation data for this assay?

We used the a non-commercial available, standardized ‘Angiostrongylus IgG Antibody Detection Kit’ produced by Shenzhen Kangbaide Biotech Company (Shenzhen, People’s Republic of China). The cut-off value (CoV) for a positive result was calculated as 2.1 standard deviations above the mean OD value of the negative control (mean + 2.1 S.D.), with a baseline value of 0.10 used if the negative control OD was ≤ 0.10. The test was considered invalid if the negative control OD exceeded 0.15, or if the positive control OD was less than 0.50. This has now been added to the method section, please see lines: 170-196.

- How many weeks or months after the onset of symptoms was serum drawn for ELISA testing? Please add the mean and range (in days) for all samples tested by this method.
Unfortunately, the time of when patient serum was taken was not systematically recorded during the study. We acknowledge the importance of such data in interpreting ELISA results, as the timing of serum collection relative to symptom onset can influence antibody levels. Instead, patients were tested (one time) after entering the hospital, if there was suspicion of *Angiostrongylus* infection. This limitation has now been mentioned in the method and discussion, please see lines: 194-196 and 463-467.

- What positive and negative controls were employed in the CO1 and ITS2 PCRs?
Two PCR controls were used for each run, a negative control containing water, and a positive control containing DNA extracted from a laboratory-strain of *[Angiostrongylus cantonensis](https://cn.bing.com/search?q=angiostrongylus+cantonensis&FORM=AWRE).* This has now been added to the method section, please see lines: 216-217.

Lines 121-122: Please briefly summarise what diagnostic methods are recommended in the PRC Health Industry Standards (WS321-2010), so that readers understand exactly what diagnostic methods were employed in the cases reported.

The PRC Health Industry Standards (WS321-2010) manual outlines the diagnostic tests required to confirm the clinical diagnosis of both suspected and confirmed cases of human angiostrongyliasis in patients admitted to hospitals. It details the procedures for performing and interpreting blood count tests, immunological tests (e.g., ELISA), and the necessary steps to examine a patient’s cerebrospinal fluid, eyes, brain tissue, and pulmonary arteries for the presence of *Angiostrongylus cantonensis* infection. This has now been added to the method section, please see lines: 161-166.

Line 148: Was Sanger sequencing used?

Yes, Sanger sequencing in the forward direction was used for both the COI and ITS2 genes fragments. This has now been mentioned in the method section, please see lines: 224-225.

Lines 154-156: How many bootstraps were used in the neighbour joining tree.

We have now redone our phylogenetic analysis. We have now replaced our Neighbour Joining tree with a Maximum Likelihood tree, using a general time reversible model with gamma rate correction (GTR+Γ). We performed this in MEGA 11, with bootstrap analysis undertaken using 1000 replicates. Please see lines: 230-233 and the new Figure 4.

Lines 162-171: What were the odds ratios of male vs female for confirmed angiostrongyliasis cases? What about the chi-squared or Fisher exact test analysis of age brackets (0-5 years old, 5-10 years old, 10-15 years old, >15 years old)? Odds ratio for specific employment types? Odds ratio of eating specific high-risk foods? Please perform more detailed statistical analyses to determine if any of the reported factors were statistically significant.

Unfortunately, the patient data presented in our study was observational, and we are unable to test for significant differences in gender, age, employment types, or behaviors of the reported patients due to the absence of a control group for comparison. We acknowledge the importance for more detailed statistical analyses, but without a control group, we cannot perform a Chi-squared test, Fisher’s exact test, or calculate the odds ratios. This limitation has now been mentioned in the discussion section, please see lines: 454-457.

Reviewer #2: The authors present a very interesting article detailing the cases of angiostrongyliasis diagnosed in their area from 2008-2021 along with testing of snails for this pathogen. Many of the cases are from an outbreak of the parasite that occurred in 2007-2008.

They reviewed all human cases of angiostrongyliasis in their Prefecture, although they do not include their selection criteria for cases or how cases were identified. The also screened snails in wet markets with monthly testing.

We had no selection criteria for cases. All patients included in this study sought medical care independently, presenting themselves to hospitals in the Dali Prefecture due to illness. The diagnoses of Angiostrongylus cantonensis infection were subsequently confirmed by the respective hospitals. This has now been clarified in the method section, Please see lines: 147-150.

Reviewer #3: Yes, good study design and a very comprehensive study.

-----

Results:

Reviewer #1:

- Does the analysis presented match the analysis plan? Yes
- Are the results clearly and completely presented? No
- Are the figures (Tables, Images) of sufficient quality for clarity? No

I cannot find figure legends anywhere in the manuscript – please include these. The figure legend for figure 4 should include what method and software was used to prepare the tree, and how many bootstrap replicates were performed. I would like to review the details of the legends once they have been included in the manuscript.
Apologizes, this was a mistake when uploading the figure files. We have now added the figures and tables (with legends) into the manuscript directly. The phylogenetic trees were created using MEGA 11, we have now mentioned this in the method section (lines: 230-233). Please see the new figure and tables.

Please change the x axis (months) labels in figures 1b and 3b from numbers to abbreviated month names (e.g. Jan, Feb, Mar, etc.)

This has now been done, please see the updated figures 1b and 3b.

The data provided in Figure 4 (the phylogenetic tree) is insufficient. Some data from the supplementary data table should be included here after the Genbank accession numbers. Please include: - city of isolation, - date of isolation, - source (Pomacea spp., human, etc.). Note that the outlier is *Caenorhabditis elegans*. A second outlier which is phylogenetically closer to A. cantonensis should be included also, I suggest - *Aelurostrongylus abstrusus,* or *Angiostrongylus vasorum*.
We have now redone the tree with your suggested edits. Please see the new updated Figure 4 and figure legend.

Please also add more sequences from global *A. cantonensis* isolates to figure 4 to ensure that the Yunnan genotypes can be visualised in the context of global genotypes. I suggest adding at least a sequence of *A. cantonensis* from each continent (North America, South America, Europe, Africa, Australia-Pacific, somewhere in Asia other than China).

We have now put more sequences from NCBI that were a match for our COI and ITS gene fragments. We were able to include sequences from the Americas, Europe, Asia and Australia-Pacific. Please see the updated Figure 4 and figure legend.

Table 1: Please add the number (n) and age (mean average and range) of patients from which samples were included in this analysis in the table legend.
We have now included this in the new Table 1. Please see the new Table 1.

Please add the median value and range of indices reported in table 1
We have now included median and range values in the new Table 1. Please see the new Table 1.

Please add units for all indices reported in table 1. Use S.I. units: https://www.nist.gov/pml/owm/metric-si/si-units
We have now included S.I. units in the new Table 1. Please see the new Table 1.

For precision of analysis, peripheral eosinophil count in Table 1 should be reported as whole numbers and not as a percentage of the white cell count. The generally accepted normal range for eosinophils is ≤0.5 x10^9 cells/L. Please re-analyse your data using an internationally recognised normal eosinophil range in cells/L.

We have redone our analysis and now added ‘Absolute Eosinophils (×10^9^/L)’ to Table 1 as suggested. Please see the new Table 1.

Lines 188-189: 87.5% of patients had peripheral eosinophilia and only 34% of patients exhibited leukocytosis. Please explicitly state how many patients had normal FBC results (no leukocytosis or eosinophilia).
We have now clarified how many patients had normal eosinophil and leukocyte levels. Please see lines: 309-310.

Lines 210-212. Please expand on the seasonality observed in snail infections. What is the climate like in these months in the area the snails come from? Winter? Summer? Wet? Dry? Describe this and please consider how this might affect rat and snail behaviour and populations, then provide some suggestions as to why these months saw such a spike in mollusk infections. Could seasonal variation in food availability (and consequent abundance) and degree of activity of reservoir host rats influence the seasonality of intermediate host of snail infections from source regions? Could seasonal variability in the abundance and activity of intermediate host snails influence the seasonality of intermediate host of snail infections from source regions?

We have now expanded upon on the seasonality change in infection for the snails. However, the snails purchased from the Dali market have now history of where they were from. They are likely sourced from a number of different locations, making it harder to determine the sudden increase. Please see lines: 366-378.

Reviewer #2: The authors present clinical and demographic data for the 125 cases of Angiostrongylus infection they identified, along with the percent of snails tested that were positive for the parasite.

Reviewer #3: Yes, very well-presented and well-written.

Conclusions:

Reviewer #1:

- Are the conclusions supported by the data presented? Yes
- Are the limitations of analysis clearly described? No
- Do the authors discuss how these data can be helpful to advance our understanding of the topic under study? Somewhat - more detail could be included
- Is public health relevance addressed? Somewhat - more detail could be included

This manuscript's greatest value is the large number of angiostrongyliasis cases identified, as such a large cohort is difficult to obtain, so the clinical indices data is very useful. This should be highlighted more. As you do not have molecular results (CSF PCR) confirming that all of your cases are truly angiostrongyliasis, how can you be sure that the diagnoses are correct? Please discuss this limitation thoroughly.

As this study began in 2007, PCR techniques were not widely available in hospitals in Dali. It was only in the past six years that PCR methods became more commonly used in diagnostic settings. While we have conducted trials using PCR detection methods on human cerebrospinal fluid, detecting *Angiostrongylus* infections in humans using PCR techniques (e.g., rtPCR or qPCR) with blood or cerebrospinal fluid remains challenging. These techniques are still in the early stages of development and testing, with a risk of false negatives. As noted in this study, the number of “suspected cases” has decreased to zero since 2011, reflecting improvements in diagnostic techniques at hospitals in Dali. We have now added this point to the discussion. Please see lines: 446-453 and 467-474.

What were the limitations of the study performed? Please briefly discuss these.

Our study has several limitations due to the design of the study and the nature of disease. We have now discussed these limitations, please see lines: 446-474.

Why were only Pomacea spp. snails sampled? Are these the only snails or mollusks sold for consumption in Dali? Please explain the choice of Pomacea spp. only in the manuscript.
As mentioned in lines: 536-539, approximately 75% of the 334 cases reported before 2006 were directly related to the consumption of *Pomacea spp.* (Lv et al., 2011). *Pomacea spp.* snails are also commonly sold and consumed in Dali, while other popular edible snails such *Achatina fulica* were are not commonly sold in the Dali markets. We have now addressed this in lines: 539-542.

Lv S, Zhang YI, Steinmann P, Yang GJ, Yang KU, Zhou XN, Utzinger J. The emergence of angiostrongyliasis in the People’s Republic of China: the interplay between invasive snails, climate change and transmission dynamics. Freshw Biol. 2011;56:717-734.

Sampling only two food stalls is not a valid epidemiological survey of snails. While the data is worth including, the epidemiological limitations of this sampling approach must be discussed.
Only two stalls consistently sell Pomacea snails at the market we investigated. We made an agreement with the vendors for them to supply us with their stock monthly. We have now added this limitation to the discussion. Please see lines: 458-463.

Line 316-320: The statement made in this section is untrue. The CDC in the United States runs a real time PCR for A. cantonensis on CSF and a positive result is a definitive indicator if A. cantonensis central nervous system infection. This must be acknowledged and discussed.

Refer to: Qvarnstrom Y, Xayavong M, da Silva AC, Park SY, Whelen AC, Calimlim PS, Sciulli RH, Honda SA, Higa K, Kitsutani P, Chea N. Real-time polymerase chain reaction detection of Angiostrongylus cantonensis DNA in cerebrospinal fluid from patients with eosinophilic meningitis. The American journal of tropical medicine and hygiene. 2016 Jan 1;94(1):176.

This section has now been rewritten, and we have now added this point to the discussion. Please see lines: 467-474 and 525-532.

Reviewer #2: The authors noted a clear seasonality for when cases seem to occur each year, with most cases in February - April. They also provide interesting data regarding diagnostic testing for angiostrongylus, which shows the widely varying utility of varying diagnostic tests.

Reviewer #3: Yes, very comprehensive and an important study

------

Editorial and Data Presentation Modifications?

Reviewer #1:

Line 251: Why only freshwater snails? Terrestrial snails, slugs and semi-slugs are all intermediate hosts of A. cantonensis, why were these not included. What about public health control of rats?
Pomacea snails are most important as they are highly compatible with A. cantonensis infection and are commonly sold/consumed in Dali. Semi-slugs and slugs are not eaten in Dali, while other edible land snails such as *Lissachatina fulica* are not commonly sold or consumed. Rat control is also an important factor and has now been mentioned. This section has now been rewritten. Please see lines: 435-443.

Line 256: I have not previously encountered the term “Guangzhou roundworm” referring to A. cantonensis. Please add the common name (rat lungworm) in brackets after this name.

This has now been changed to *A. cantonensis*. Please see line: 443.

Line 351: It is not really imperative that this investigation be performed, please soften this wording.

We have now changed this. Please see lines: 564-566.

Please add further discussion on the role of rats in the A. cantonensis life cycle to the introduction.

This has now been added. Please see lines: 70-78.

Please write “*Pomacea spp.*”, not just “*Pomacea*” throughout

We have now changed this. Please see the changes throughout the new manuscript.

Note that while in entomology and malacology often the first two letters of a genus are included in abbreviation, in parasitology this is not done. Please change all “An. cantonensis” to “A. cantonensis” throughout the manuscript.
This had been changed throughout the manuscript.

Some of the epidemiological discussion in the manuscript is unnecessarily verbose and could be rewritten in a more succinct manner.

We have now rewritten our discussion to be more succinct. Please see lines: 478-479, 484-496, 523-532, 552-553, 560-561 and 564-566.

A short concluding paragraph summarising the most important findings of this study is necessary.
We have included a conclusion at the end of the discussion. Please see lines: 567-572.

The title could be shortened to something like, “Monitoring the trends of Angiostrongylus cantonensis in humans and Pomacea snails, Dali, Yunnan, China, 2007-2021.

We have changed the title to your suggestion. Please see the new title.

Reviewer #2: (No Response)

Reviewer #3: (No Response)

-----

Summary and General Comments:

Reviewer #1: This report summarises data on human cases of angiostrongyliasis from 2007 to 2021 in Yunnan, China. The high number of cases described gives this report merit, as larger scale clinical data such as this is difficult to obtain. The survey of gastropods from food sellers is adjunct, and also has merit. My major criticisms are that additional details of the methods employed are required, as well as ranges for the reported clinical data. I have further, more minor comments, detailed below.

The authors may wish to follow up on IgG serology for these patients in another study. A major gap in our understanding of A. cantonesis diagnostics is how many patients mount an IgG antibody response, and how long this response lasts for. A longitudinal study of A. cantonesis IgG titres in a large cohort such as this would be very helpful for clinical diagnostics and surveillance purposes.

Thank you for your suggestions. We also agree that a further study with a focus on IgG serology in future patients would be help better understand the IgG antibody response and the duration of the response during infection. Hopefully, a future cohort study can be designed with the experience gained from this study.

Reviewer #2: Overall, this was an interesting and well written paper that presents useful information regarding the presentation of a generally neglected parasitic infection, Angiostrongylus.

Reviewer #3: A very substantial study and well-presented. I have only minor mostly grammatical suggestions outlined below.

Over a very well-written and significant paper. This is one of few papers that documents snail infection rates and corroborates it with human infection temporally (over an extensive time period). I have only minor suggestions outlined below. A question I have, and likely not possible with this large dataset, is if there are subsets of human samples available to run PCR? Also, I noticed the annealing temperature used for PCR of snails is very low. Is there a reason for this? I am assuming cross-reactivity would be picked up by sequencing, so I am wondering if the annealing temperature was evaluated, and how was 48C selected. The authors might want to include a statement in the methods. Also, I was a bit surprised of the relatively low infection rates in snails.

This study in started in 2007, when PCR and sequencing were not widely available in the Dali hospitals. Only until 6 years ago were PCR methods widely available for use in the diagnostic hospitals in Dali. We have done some trials using PCR detection methods on human cerebrospinal fluid before, but we find detecting *Angiostrongylus* infection in humans using PCR methods (e.g. rtPCR or qPCR) with blood or cerebrospinal fluid is difficult. The technique is still in the early stages of development and testing with a chance of false negatives at present. We have discussed this limitation in our discussion. Please see lines: 467-474.

L30: analyze -
L56: gastropod -
1) L57: A. cantonensis not An. cantonensis?
L60: Might want to include Hawaii -
L98: outline -
2) L101: Is the ethics statement properly placed within the manuscript?
3) L145: 48C seems really low. All products were sequenced?
L161: cases
L193: content
L206: In total, 49,970 …..
L239: collect Pomacea snails and eggs.
L281: different from
4) L316-318: Why not use PCR of CSF or blood as a confirmatory test?

Thank you, all of your suggested edits have been done in the new manuscript.

Please note:

1. This had been changed throughout the manuscript.
2. The Ethical statement was requested by the Journal to be the first section of the Material and Methods.
3. The Annealing temperature for both the COI and ITS was 50°C, but for problematic samples, a low-stringency PCR was performed, wherein the annealing temperature was reduced to 48°C. All PCR products were sequenced and confirmed to be A. cantonensis. This has now been clarified (Lines: 216-225).
4. Please see the reply above. We have now added this point to the discussion, please see lines: 467-474.

-----

Do you want your identity to be public for this peer review? For information about this choice, including consent withdrawal, please see our [Privacy Policy](https://track.editorialmanager.com/CL0/https://www.plos.org/privacy-policy/1/010f01935ddeba40-ec8b8b6e-739c-4672-98cb-58147123d523-000000/TmB6wKoMwA1FDYT2ZwtvQnFatruIwG-MMkwgQUwyrh4=186).

Reviewer #1: Yes: Richard Bradbury

Reviewer #2: No

Reviewer #3: No
