# Supplementary material for: Monitoring the trends of Angiostrongylus cantonensis infection in humans and Pomacea spp. Snails in Dali, Yunnan, China, 2007–2021
Source: PLoS Negl Trop Dis. 2025 May 5;19(5):e0013065. doi: 10.1371/journal.pntd.0013065 (PMC12052120; doi:10.1371/journal.pntd.0013065)
Supplement: Attachment — Submitted filename: PNTD-D-24-01132R1 rev3 comments.docx [file pntd.0013065.s004.docx]

Manuscript: **PNTD-D-24-01132R1**

**Reviewer 3 comments**

L79-80 Aren’t all human angiostrongyliasis cases caused by A. cantonensis?

L210 Why did you use ITS2 instead of ITS1?

L319-321 Aren’t abnormal shadows in lungs abnormal results? What’s the difference?

L415 Both trees were…

L441-443 This sentence sounds like the local residents do consume raw or undercooked gastropods to intentionally interrupt the transmission cycle, do they?

L465 is significant

L472-474 PCR-based diagnostics have been available for A. cantonensis for nearly 10 years. PCR is run optimally on CSF, but also can use blood.

L491-493 Was there an increase in rainfall during these times?

L530-532 real-time PCR is not a ‘new’ diagnostic method, and should be considered as standard methodology for detection of A. cantonensis. It can be detected more often in the CSF, but can also be detected in the blood.

L535 have become the main intermediate hosts
