# Supplementary material for: Monitoring the trends of Angiostrongylus cantonensis infection in humans and Pomacea spp. Snails in Dali, Yunnan, China, 2007–2021
Source: PLoS Negl Trop Dis. 2025 May 5;19(5):e0013065. doi: 10.1371/journal.pntd.0013065 (PMC12052120; doi:10.1371/journal.pntd.0013065)
Supplement: Attachment — Submitted filename: Response to Reviewers 24-03.docx [file pntd.0013065.s006.docx]

PNTD-D-24-01132R1

Monitoring the Trends of Angiostrongylus cantonensis Infection in Humans and Pomacea spp. Snails in Dali, Yunnan, China, 2007-2021

PLOS Neglected Tropical Diseases

Dear Dr. Guo,

Thank you for submitting your manuscript to PLOS Neglected Tropical Diseases. After careful consideration, we feel that it has merit but does not fully meet PLOS Neglected Tropical Diseases's publication criteria as it currently stands. Therefore, we invite you to submit a revised version of the manuscript that addresses the points raised during the review process.

Please submit your revised manuscript within 30 days Apr 22 2025 11:59PM. If you will need more time than this to complete your revisions, please reply to this message or contact the journal office at plosntds@plos.org. When you're ready to submit your revision, log on to https://www.editorialmanager.com/pntd/ and select the 'Submissions Needing Revision' folder to locate your manuscript file.

Please include the following items when submitting your revised manuscript:

* A rebuttal letter that responds to each point raised by the editor and reviewer(s). You should upload this letter as a separate file labeled 'Response to Reviewers'. This file does not need to include responses to any formatting updates and technical items listed in the 'Journal Requirements' section below.

* A marked-up copy of your manuscript that highlights changes made to the original version. You should upload this as a separate file labeled 'Revised Manuscript with Track Changes'.

* An unmarked version of your revised paper without tracked changes. You should upload this as a separate file labeled 'Manuscript'.

If you would like to make changes to your financial disclosure, competing interests statement, or data availability statement, please make these updates within the submission form at the time of resubmission. Guidelines for resubmitting your figure files are available below the reviewer comments at the end of this letter.

We look forward to receiving your revised manuscript.

Kind regards,

Siddhartha Mahanty, M.B.B.S., M.P.H

Academic Editor

PLOS Neglected Tropical Diseases

Additional Editor Comments :

The reviewers noted that the manuscript has been considerably modified and improved to address their main concerns but there are some less critical changes recommended by them that, if addressed, would raise the quality of the paper considerably. Please address the issues mentioned in their summary comments and in an attachment from Reviewer 3. Please respond to these issues for the manuscript to be acceptable for publication in the Journal.

Comments to the Authors:

Please note that the reviews are uploaded as attachments.

Reviewers' comments:

Reviewer's Responses to Questions

Key Review Criteria Required for Acceptance?
As you describe the new analyses required for acceptance, please consider the following:

Methods:

-Are the objectives of the study clearly articulated with a clear testable hypothesis stated?
-Is the study design appropriate to address the stated objectives?
-Is the population clearly described and appropriate for the hypothesis being tested?
-Is the sample size sufficient to ensure adequate power to address the hypothesis being tested?
-Were correct statistical analysis used to support conclusions?
-Are there concerns about ethical or regulatory requirements being met?

Reviewer #1: (No Response)

Reviewer #3: Yes, but no comment on the statistical methods.

---

Results:

-Does the analysis presented match the analysis plan?
-Are the results clearly and completely presented?
-Are the figures (Tables, Images) of sufficient quality for clarity?

Reviewer #1: (No Response)

Reviewer #3: It's much improved

---

Conclusions:

-Are the conclusions supported by the data presented?
-Are the limitations of analysis clearly described?
-Do the authors discuss how these data can be helpful to advance our understanding of the topic under study?
-Is public health relevance addressed?

Reviewer #1: (No Response)

Reviewer #3: Yes, again much improved

---

Editorial and Data Presentation Modifications?

Use this section for editorial suggestions as well as relatively minor modifications of existing data that would enhance clarity. If the only modifications needed are minor and/or editorial, you may wish to recommend “Minor Revision” or “Accept”.

Reviewer #1: (No Response)

Reviewer #3: Minor revision, I don't need to see again if my suggested changes are made.

---

Reviewer #1: The authors have satisfactorily addressed all of my review comments.

Note on taxonomic writing: Throughout the revised version, "spp." is italicised (i.e. "Pomacea spp."). Only full Latin and Greek genus/species names need to be italicised. As this is an abbreviation of an English word ('species"), it should not be italicised.

Thank you, we have now changed all ‘spp.’ to be unitalicised. Please check throughout the new manuscript.

Reviewer #3:

I believe the authors adequately addressed the reviewer's concerns.

1. Is there no way to retrieve the dates of blood draw? This would significantly strengthen this ms.

Unfortunately, as mentioned previously, due to the age and the retrospective nature of the data, we are unable to retrieve the dates of patients’ blood draws, as the exact timing of serum collection was not systematically recorded by the hospitals. We acknowledge the importance of this information and will ensure that future studies include such patient details in future.

2. L79-80 Aren’t all human angiostrongyliasis cases caused by A. cantonensis?

Yes, *A. cantonensis* causes the majority of human cases. However, *A. costaricensis* causes abdominal angiostrongyliasis, and in theory, *A. malaysiensis* can be pathogenic to humans also. We have now mentioned this in the introduction, please see lines: 81-84.

3. L210 Why did you use ITS2 instead of ITS1?

Our laboratory prefers using the ITS2 region because it demonstrated higher rates of amplification with low DNA quantities and greater specificity for *A. cantonensis*. In our experience, the ITS2 primers outperform ITS1 primers for detecting the parasite in field-collected snail samples. However, we acknowledge the broader popularity of ITS1 primers and will consider their use in future studies.

4. L319-321 Aren’t abnormal shadows in lungs abnormal results? What’s the difference?

Yes, these head CT results are abnormal, we have now re-worded this to make this clearer. Please see lines: 321-323.

5. L415 Both trees were…

Thank you, this has now been corrected. Please see line: 415.

6. L441-443 This sentence sounds like the local residents do consume raw or undercooked gastropods to intentionally interrupt the transmission cycle, do they?

We have now re-written this section to make it clearer. This region of China has culinary practices that includes consuming seasoned snails, and prawns (as mentioned later on in lines 476–478). Please see lines: 441–445.

7. L465 is significant

This has now been corrected. Please see line: 467.

8. L472-474 PCR-based diagnostics have been available for A. cantonensis for nearly 10 years. PCR is run optimally on CSF, but also can use blood.

This has now have now re-written and has been corrected. Please see line: 474-476.

9. L491-493 Was there an increase in rainfall during these times?

Yes, on average the Dali region does experience an increase in rainfall during the spring months. However, as mentioned previously, the location and origins of the market sold Pomacea spp. are unknown. So the increased rainfall in the Dali region, may not be relevant to why these particular market snails had an increase in infection (as mentioned in lines: 365-374).

10. L530-532 real-time PCR is not a ‘new’ diagnostic method, and should be considered as standard methodology for detection of A. cantonensis. It can be detected more often in the CSF, but can also be detected in the blood.

As this study started in 2007, real-time PCR was not standard methodology for detection of *A. cantonensis*. In the last 10 years, it has definitely become more popular, but still remained not widely used in rural areas of South Asia and South East Asia until more recently (when compared to America). This section has now been re-worded and corrected as suggested. Please see line: 531-536.

11. L535 have become the main intermediate hosts

This has now been corrected. Please see line: 539.
